# Supplementary material for: Retrospective study: clinicopathological features and prognosis of idiopathic membranous nephropathy with seronegative anti-phospholipase A2 receptor antibody
Source: PeerJ. 2020 Feb 21;8:e8650. doi: 10.7717/peerj.8650 (PMC7039122; doi:10.7717/peerj.8650)
Supplement: Supplemental Information 3 [file peerj-08-8650-s003.docx]

**File 1**

**In column D Sex:** “1” equivalent to “male”, “0” equivalent to “female”.

**In column G Urine Protein:** “1” equivalent to “urine protein +”, “2” equivalent to “urine protein ++”; “3” equivalent to “urine protein +++”; “4” equivalent to “urine protein ++++”,

**In column AE Group:** “1” equivalent to “anti-PLA2R antibody negative”, “2” equivalent to “anti-PLA2R antibody positive”.

**In column AL/AM/AN/AO/AP/AQ Tissue IgG/IgM/IgA/C3/C1q/Fib:** “0” equivalent to “-”; “1” equivalent to “+”; “2” equivalent to “++”; “3” equivalent to “+++”.

**In column AU/AV/AW/AX/AY/AZ/BA/BB/BC Global sclerosis/ Glomerular mesangial hyperplasia/ Acute renal tubular lesions/ Chronic renal tubular lesions/ Inflammatory cell infiltration/ vascular disease/ crescents/ hyperplasia endothelialis/ Balloon adhesion:** “1” equivalent to “The patient's kidney biopsy revealed corresponding pathological manifestations”;

**File 2**

**In column C Sex:** “1” equivalent to “male”, “0” equivalent to “female”.

**In column F Urine Protein:** “1” equivalent to “urine protein:+”, “2” equivalent to “urine protein :++”; “3” equivalent to “urine protein:+++”; “4” equivalent to “urine protein:++++”,

**In column AD Group:** “1” equivalent to “anti-PLA2R antibody negative”, “2” equivalent to “anti-PLA2R antibody positive”.

**In column AK/AL/AM/AN/AO/AP** **Tissue IgG/IgM/IgA/C3/C1q/Fib:** “0” equivalent to “-”; “1” equivalent to “+”; “2” equivalent to “++”; “3” equivalent to “+++”.

**In column AT/AU/AV/AW/AX/AY/AZ/BA/BB Global sclerosis/ Glomerular mesangial hyperplasia/ Acute renal tubular lesions/ Chronic renal tubular lesions/ Inflammatory cell infiltration/ vascular disease/ crescents/ hyperplasia endothelialis/ Balloon adhesion:** “1” equivalent to “the patient's kidney biopsy revealed corresponding pathological manifestations”;

**In column BH TURINE:** “1” equivalent to “urine protein: +”, “2” equivalent to “urine protein: ++”; “3” equivalent to “urine protein: +++”; “4” equivalent to “urine protein:++++”;

**In column BO CANCER:** “1” equivalent to “the patient had a tumor”; “0” equivalent to “the patient did not develop a tumor”;

**In column BP diabetes:** “1” equivalent to “the patient had diabetes”; “0” equivalent to “the patient did not have diabetes”;
